# Health co-benefits of sustainable dietary transitions to reduced red and processed meat intake in the United Kingdom: a modelling study

**DOI:** 10.1007/s00394-026-04093-6

**Published:** 2026-09-17

**Authors:** Patricia Eustachio Colombo, Olivia Auclair, James Milner, Angela Fontan, Silvia Pastorino, Sara Monteiro Pires, André Hesselink, Marie Löf, Rosemary Green

**Affiliations:** 1 Department of Medicine, Karolinska Institutet, 17177 Stockholm, Sweden; 2 Centre On Climate Change and Planetary Health, London School of Hygiene & Tropical Medicine, Keppel St, London, WC1E 7HT UK; 3 Environmental Change Institute, School of Geography and the Environment, University of Oxford, 3 S Parks Rd, Oxford, OX1 3QY UK; 4 Department of Public Health, Environments and Society, London School of Hygiene and Tropical Medicine, 15-17 Tavistock Place, London, WC1H 9SH UK; 5 Division of Decision and Control Systems, KTH Royal Institute of Technology, Malvinas V 10, 10044 Stockholm, Sweden; 6 National Food Institute, Technical University of Denmark, Kemitorvet Building 201, 2800 Kgs Lyngby, Denmark; 7 Department of Internal Medicine and Clinical Nutrition, Sahlgrenska Academy, University of Gothenburg, 40530 Gothenburg, Sweden

**Keywords:** Chronic disease prevention, Life expectancy, Health policy, Health impact modelling, Policy evaluation

## Abstract

**Purpose:**

Reducing meat intake and increasing plant-based food consumption are priorities for chronic disease prevention and environmental sustainability across Europe. Previous modelling has outlined how policy interventions could shift dietary patterns to align with meat reduction targets, but the resulting health impacts remain unexplored.

**Methods:**

We quantified the projected health impacts of the UK Climate Change Committee's (CCC) dietary targets—a 35% or 50% reduction in meat consumption—focusing on red and processed meat and substitution with vegetables and legumes, on incidence and mortality of key diet-related chronic diseases over 30 years. Dietary inputs were derived from an agent-based opinion dynamics model parameterised using the UK National Diet and Nutrition Survey data, and health impacts were estimated using the IOMLIFET life-table model.

**Results:**

Achieving the red and processed meat reduction components of the CCC's targets was associated with a projected increase in life expectancy of 7.2 (1.1–12.9) and 9.4 (1.3–15.4) months and years of life gained of 8.4 (1.3–14.9) and 10.9 (1.5–17.9) million, in the 35% and 50% scenarios, respectively. An estimated 3.4 (0.5–6.6) and 4.5 (0.6–8.1) million chronic disease cases could be averted. Up to 95% of health gains were attributable to cardiovascular outcomes, with three quarters linked to increased vegetable and legume consumption.

**Conclusion:**

These exploratory estimates suggest policy-driven reductions in red and processed meat aligned with climate goals could deliver substantial public health benefits. While conditional on modelled behavioural trajectories, findings offer relevant insights for European strategies to reduce cardiovascular disease burden and promote sustainable dietary transitions.

**Supplementary Information:**

The online version of this article https://doi.org/10.1007/s00394-026-04093-6 contains supplementary material, which is available to authorized users.

## Introduction

The urgent need to shift population diets toward more sustainable and health-promoting patterns is widely recognised as a cornerstone for addressing both the rising burden of chronic diseases across Europe and globally, and the escalating environmental impacts of food production and consumption [1–5]. Similar to dietary patterns observed in many European countries [6], current dietary patterns in the United Kingdom (UK) are characterised by high consumption of red and processed meat, with average weekly intakes of nearly 590 g in adult men and around 400 g in adult women [7], compared with the UK-specific recommended maximum intake of 490 g per week (70 g per day) [8]. Such high intakes have been associated with increased risks of cardiovascular disease (CVD), type 2 diabetes, colorectal cancer, and premature mortality [9], as well as with substantial environmental impacts [10]. In line with broader European climate mitigation pathways outlined by the European Commission’s roadmap for a competitive low-carbon Europe by 2050 [11], the UK’s Climate Change Committee (UKCCC) has emphasised the necessity of reducing meat consumption as part of comprehensive strategies to mitigate national greenhouse gas emissions (GHGE) and enhance public health [12]. Supporting this, epidemiological modelling studies [13–16] and the recent EAT–Lancet Commission on healthy, sustainable, and just food systems [17] suggest that a diet low in red and processed meat intake, and high in plant-based foods such as vegetables and legumes could yield substantial health and environmental co-benefits. Despite the robust evidence base that supports a transition towards a reduced consumption of meat and an increased intake of plant-based foods in high-income contexts, understanding how to reach real-world dietary change at scale remains challenging due to the complex interplay of social, cultural, economic, and behavioural factors that shape food choices [18, 19].

Agent-based and microsimulation models have emerged as powerful tools to predict the potential impact of dietary interventions and policy strategies on population-level dietary behaviours [20, 21], enabling researchers and policymakers to estimate the large-scale effects of shifts in food consumption patterns. Our previous work [22] utilised agent-based opinion dynamics models to simulate the effects of governmental influence (fiscal measures and/or information campaigns) on dietary patterns in the UK, specifically targeting reductions in meat consumption to align with the UKCCC’s climate goals [12]. This modelling demonstrated that achieving targets of 35% and 50% average meat reduction by 2030 and 2050, respectively [12], could lead to significantly increased intakes of vegetables, legumes, and meat alternatives (plant-based products such as soy- or pea-based mince, designed to replace meat in meals), alongside decreases in GHGE, land use, and water use [22]. While this prior study quantified the shifts in food group consumption and the environmental benefits, a comprehensive assessment of the associated health impacts was beyond its primary scope. Robust evidence on these health impacts is fundamental for policymakers to effectively weigh the benefits against possible drawbacks of interventions, and to foster their legitimacy and public acceptance. However, despite the recognised value of evidence-informed policymaking, research indicates that policy change in nutrition and diet is often slow and contested, facing barriers such as political priorities, industry pressure, and public scepticism [23]. According to a review by the World Health Organisation (WHO) on fiscal policies to promote healthy diets [24], including analyses focused on European settings, the provision of scientific evidence on potential benefits of new policies is critical not only to support the adoption of dietary interventions but also to sustain them amidst ongoing debate and resistance. This review also highlighted the lack of evidence on long-term health impacts as an important evidence gap to be addressed in future research.

Building on our prior work and using the UK as a case study within a wider European policy context, this study therefore aims to estimate the associated health impacts of dietary transitions towards meeting the UKCCC’s targets of 35% and 50% average meat reduction by 2030 and 2050. By quantifying the potential changes in chronic disease incidence and mortality resulting from achieving national meat reduction targets and the likely substitution to other foods this would entail, this research provides actionable insights for policymakers in the UK and other European countries seeking to promote healthier and more environmentally sustainable diets at the population level.

## Methods

### Scenarios

We employed life table modelling to quantify the health impacts resulting from previously simulated changes in meat intake in the UK [22]. Briefly, to simulate the impact of governmental influence on people’s consumption of total (red and white, processed and non-processed) meat consumption, dietary data derived from the UK National Diet and Nutrition Survey (NDNS) 2019 [7] served as the baseline input data for agent-based opinion dynamics models. Agents, representing the UK adult population, were connected within a social network and influenced through a sequence of governmental campaigns to meet the UKCCC’s targets of a 35% average reduction in meat consumption by 2030 and a 50% average reduction by 2050 [12]. While the UKCCC's dietary pathways include reduced intakes of both meat and dairy, this study focuses solely on changes in meat consumption, consistent with the upstream agent-based modelling in which only meat reduction was simulated. In a scenario of high governmental influence represented by the implementation of nationwide information campaigns for reduced meat consumption in conjunction with fiscal measures (taxes on meat + subsidies on meat alternatives/legumes/vegetables), meat consumption declined in the simulations, while the intake of vegetables, legumes, and meat alternatives increased isocalorically to offset the total simulated reduction in meat consumption, including white meat [22]. No other dietary components were assumed to change. Resulting dietary changes for each scenario are shown in Table 1. It took approximately 5.2 and 8.1 years for the 35% and 50% reduction targets to be met, respectively. Further details of the methods used for the simulations can be found in Fontan et al. 2025 [22].

**Table 1:** Changes in daily intakes for the dietary scenarios tested, based on previous work [22]

Meat reduction scenario	Males +18y					Females +18y				
	Red meat (g/day)	Processed meat (g/day)	Legumes (g/day)	Vegetables (g/day)	RR	Red meat (g/day)	Processed meat (g/day)	Legumes (g/day)	Vegetables (g/day)	RR
−35%^a^	−15.6	−14.3	+59.3	+168.9	Central	−10.4	−8.0	+35.5	+114.8	Central
−35%^b^	−27.5	−25.1	+92.3	+288.0	Lower	−19.3	−14.9	+49.2	+174.8	Lower
−35%^c^	−3.7	−3.4	+16.5	+49.8	Upper	−1.4	−1.1	+15.5	+54.8	Upper
−50%^a^	−22.5	−20.5	+83.0	+242.7	Central	−15.5	−11.9	+50.1	+171.8	Central
−50%^b^	−38.6	−35.2	+137.2	+427.6	Lower	−27.8	−21.4	+75.4	+277.3	Lower
−50%^c^	−6.4	−5.9	+19.0	+57.8	Upper	−3.2	−2.4	+18.5	+66.3	Upper

^a^The central exposure–response estimate combined with the average change in consumption

^b^The lower exposure–response estimate combined with the average change in consumption +1 standard deviation

^c^The upper exposure–response estimate combined with the average change in consumption – 1 standard deviation

RR = Relative risk estimate

The isocaloric increases in vegetables and legumes shown here were calibrated against the total simulated reduction in meat consumption (red, processed, and white combined); only the red and processed meat component of this reduction, and its associated health impacts, are modelled in the present study (see Methods)

We quantified the health impacts of meeting these two targets, specifically analysing the changes in average consumption levels of red meat, processed meat, vegetables, and legumes among adult males and females. Health impacts related to changes in meat alternatives were not modelled due to a lack of robust evidence linking their consumption to the health outcomes considered here; the existing evidence base is limited to a small number of short-term randomised trials assessing intermediate cardiometabolic risk markers rather than the disease incidence and mortality outcomes modelled in this study [25]. Health impacts of unprocessed white meat were also not modelled because evidence specifically linking white meat consumption to chronic diseases is inconclusive [26, 27]. Hence, the portion of unprocessed white meat was removed from the total simulated meat reduction proportionally to their share (36% for males and 39% for females, by weight) of total meat consumption at baseline, and was assigned no health impact in the model—representing a conservative assumption consistent with the limited epidemiological evidence for this food group. In the 35% meat reduction scenario, we quantified the health impacts associated with achieving a 35% average reduction in red and processed meat consumption within the UK population. In the 50% meat reduction scenario, we evaluated the health impacts related to a 50% reduction in red and processed meat consumption. For brevity, both scenarios are hereafter referred to as the 35% and 50% meat reduction scenarios, where 'meat' refers exclusively to red and processed meat throughout."

### Disease outcomes and relative risks

Exposure–response relationships (i.e. relative risks per unit change in consumption) between dietary intake and chronic disease morbidity and mortality were obtained from the Global Burden of Disease (GBD) study [9]. The GBD study is the most comprehensive ongoing global observational epidemiological study and has assessed 396 diseases and injuries and 87 risk factors across 204 countries since 1990. GBD-derived exposure–response functions were used to ensure internal consistency across outcomes and risk factors and to enable comparability with other global and national burden of disease assessments.

The relative risk (RR) for a given dietary risk-disease pair quantifies the change in mortality (or morbidity) risk associated with a change in exposure to that dietary risk. For example, the risk of ischaemic heart disease (IHD) is reduced by 24% for each 50 g increase in daily legume intake (Table 2). Relative risks from the GBD expressed in terms of a harmful risk factor (e.g. “diet high in red meat”) were inverted (i.e. the reciprocal of the relative risk was taken) to obtain relative risks corresponding to a beneficial dietary change. A total of six disease outcomes linked to the consumption of red meat, processed meat, vegetables, and legumes (Table 2) were considered in the model [9]. These six outcomes reflect the diet-disease pairs for which the GBD study provides relative risk estimates for these four dietary exposures, based on associations graded as convincing or probable given the strength and consistency of the underlying evidence.

**Table 2:** Dietary exposure–response pathways (including upper and lower 95% confidence intervals) used in the health impact modelling

Dietary exposure	Health outcome	Unit	Relative risk^a^	95% CI		
Vegetables	Ischaemic heart disease	100 g increase	0.86	0.78	–	0.94
	Ischaemic stroke	100 g increase	0.87	0.79	–	0.97
	Intracerebral haemorrhage	100 g increase	0.90	0.83	–	0.97
	Subarachnoid haemorrhage	100 g increase	0.90	0.83	–	0.97
Legumes	Ischaemic heart disease	50 g increase	0.76	0.65	–	0.89
Red meat	Colorectal cancer	100 g decrease	0.86	0.76	–	0.97
	Diabetes type 2	100 g decrease	0.80	0.68	–	0.97
Processed meat	Ischaemic heart disease	50 g decrease	0.56	0.39	–	0.97
	Colorectal cancer	50 g decrease	0.85	0.79	–	0.91
	Diabetes type 2	50 g decrease	0.58	0.47	–	0.76

^a^Relative risks from the Global Burden of Disease study [9]

^b^CI = Confidence interval

### Baseline health data

Age- and sex-specific data for the UK population in 2021, including population-size estimates, all-cause mortality, and disease-specific mortality (mortality rates) and morbidity (incidence rates) for relevant outcomes (Table 2) were downloaded from the GBD results tool [28].

### Health impact modelling

Health impacts from the dietary scenarios were quantified following a previously developed approach [29, 30] using the IOMLIFET life table model [31] implemented in R version 4.5.1 [32]. This model projects population survival patterns by applying changes in mortality risk from hypothetical dietary changes to age-specific mortality rates. By inputting hypothetical dietary changes (representing altered risk exposures) and applying known exposure–response functions (i.e. relative risks), the model quantifies subsequent changes in life expectancy and years of life lost (mortality outcomes) across time. Years of life lost represent the years of life lost for an individual (or a population) as a result of premature avertable mortality, considering the age at which deaths occurred. Since the dietary modifications were expected to reduce mortality rates, years of life lost were translated to years of life gained (YLG). Changes in morbidity (new cases of disease) were quantified using the same principles. Morbidity calculations used the life table population output as the baseline, to which changes in risk exposures were applied. Each morbidity calculation was performed independently based on incidence rates and then aggregated across the entire UK adult population. Changes in YLG and disease incidence were aggregated over a 30-year period. This duration was selected to allow the full observation of latent health impacts, as discussed further below. Changes in life expectancy at birth were determined by calculating the difference between the baseline life expectancy (total expected life years divided by the initial population) and the modelled life expectancy (impacted expected life years divided by the impacted starting population) across the full health projection.

Our modelling strategy incorporated several key assumptions regarding dietary changes and their health outcomes (Supplementary Table 1). Where multiple dietary exposures influenced the same disease outcome, relative risks were multiplied together to avoid double counting of overlapping pathways. This is equivalent to summing the natural logarithms of the relative risks and exponentiating the result, and follows standard comparative risk assessment methodology for combining the effects of multiple dietary risk factors. This approach assumes that the proportional effect of each dietary exposure on disease risk is independent of the levels of the other exposures considered; because vegetable, legume, and meat intake changed concurrently within each scenario by design, this assumption could not be empirically tested in the present study, although it follows the same approach used in the underlying GBD risk factor framework. To account for the time required for dietary changes to manifest as health impacts, we integrated time lags based on established epidemiological evidence [33, 34] and a previously applied modelling approach [35]. Specifically, we assumed that the time to full effect (TTFE) on all disease outcomes was approximately 10 years in the main analyses. More information regarding data management and assumptions for the health impact modelling may be found in the supplementary materials (full list of assumptions in Supplementary Table 1; lag functions in Supplementary Fig. 1).

### Sensitivity analysis

To assess the sensitivity of our results to key parameters, we generated upper and lower health impact estimates (i.e. confidence intervals) for the modelled outcomes. These were based on a dispersion measure of ±1 standard deviation around the mean for changes in the consumption of red meat, processed meat, vegetables and legumes. These bounds reflect variability in baseline dietary intake across individuals in the NDNS sample, rather than uncertainty arising from the agent-based simulation itself. These estimates were combined with the upper and lower 95% Confidence Intervals (CIs) for the RRs provided by the GBD study [9] (Table 2). In addition, we explored different lags between reduction in disease risk factors and potential health gains, as per previous health impact modelling research [35]. The time perspective for health gains varies by disease: cardiovascular disease mortality may respond within a few years following risk factor reduction, while cancer outcomes are associated with longer exposure durations [35]. To balance these time perspectives while accounting for the morbidity burden of both disease groups, we followed the approach of Fadnes et al. in assuming a TTFE of 10 years with a gradual, linear increase in effect as the main analysis. Sensitivity analyses with 5 and 30 years to full effect were also conducted to reflect this uncertainty [35].

## Results

**Fig. 1:**
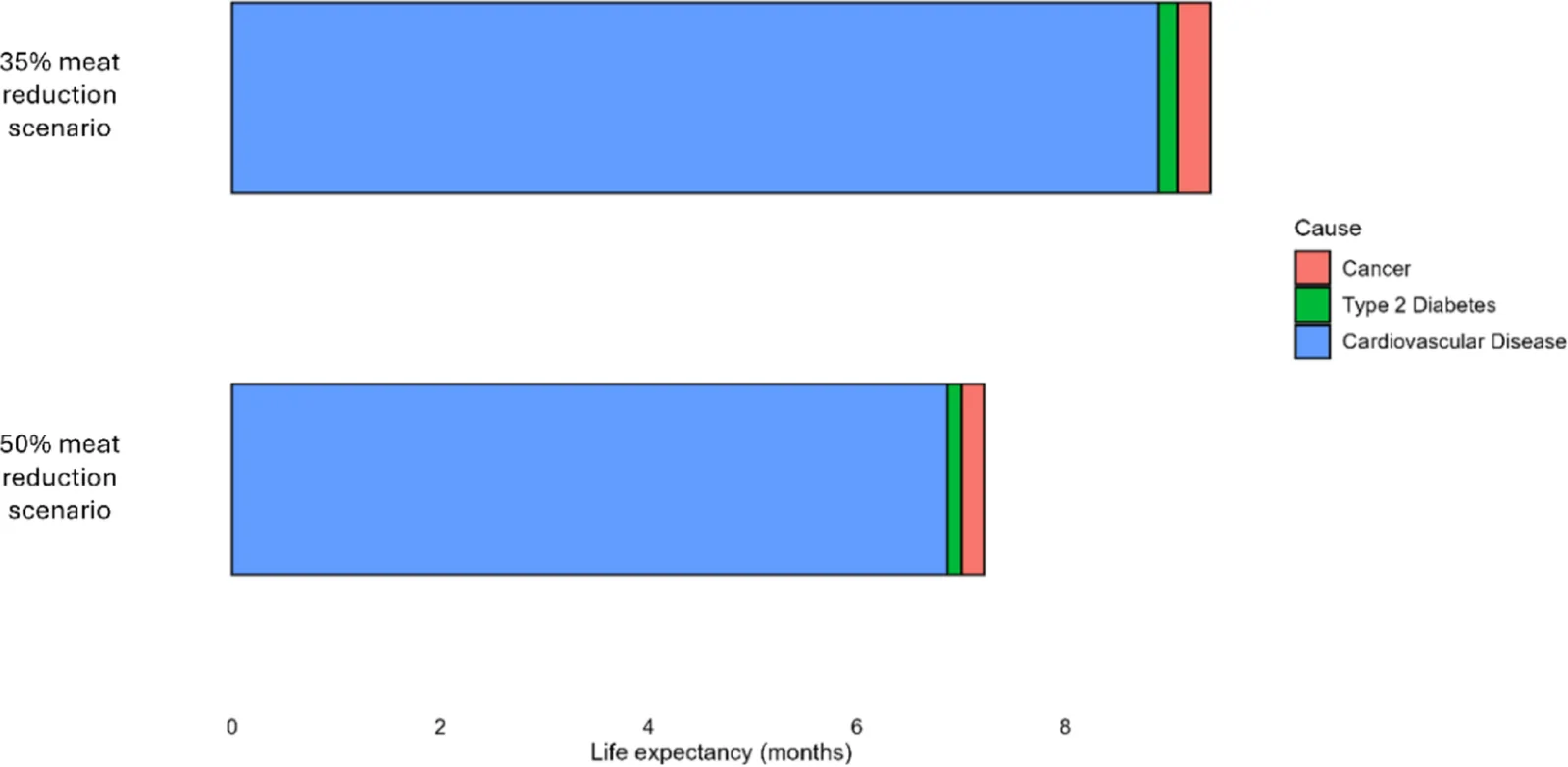
Share of change in life expectancy by disease outcome across the health impact projection for the 35% and 50% meat reduction scenarios, respectively

**Fig. 2:**
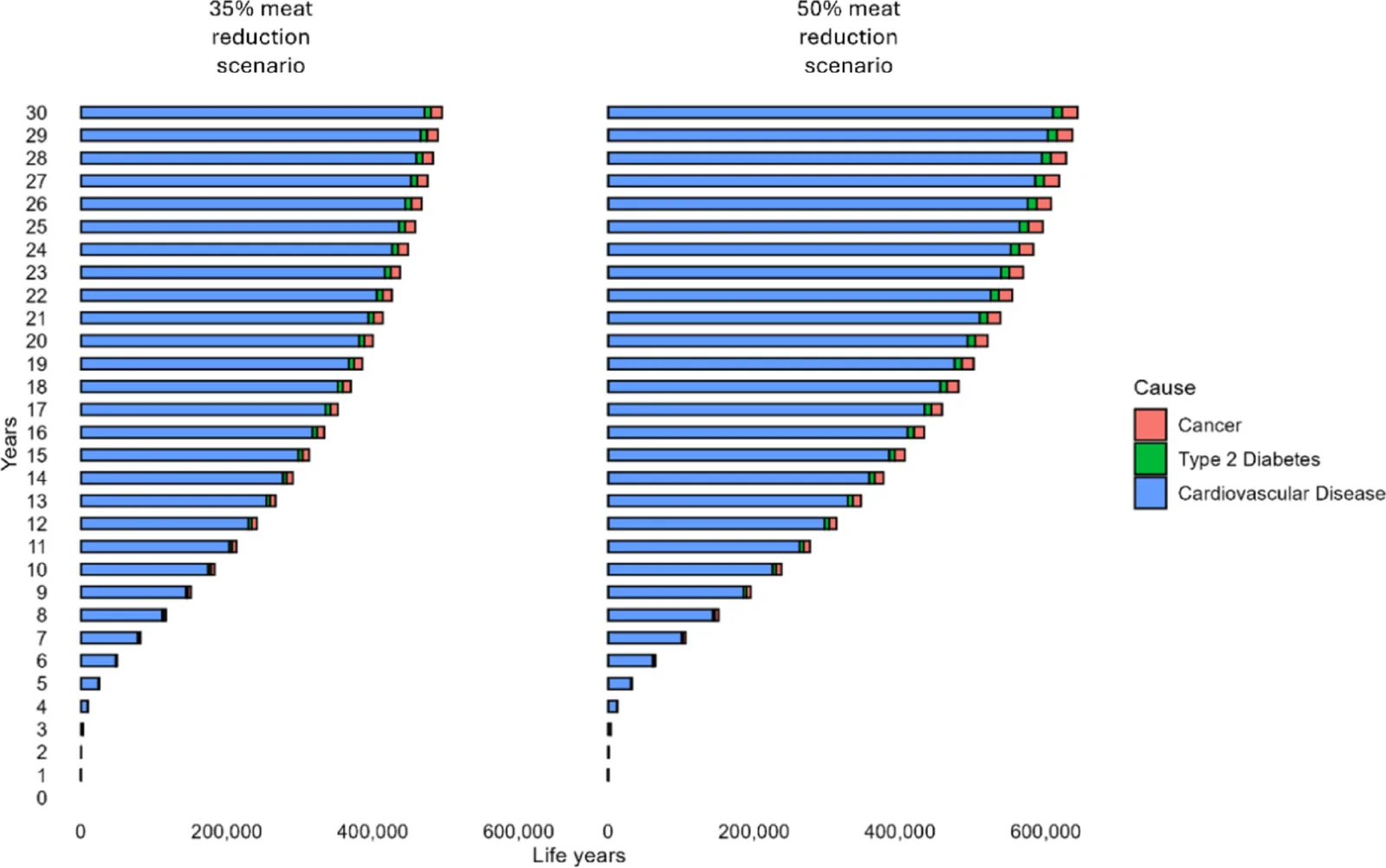
Share of changes in years of life gained by disease outcome, with a time to full effect of 10 years, for the 35% and 50% meat reduction scenarios, respectively

The health impact modelling results indicated that reaching the UKCCC’s meat reduction targets would have positive impacts on average life expectancy in the UK. Specifically, life expectancy would increase by 7.2 (1.1–12.9) months in the 35% meat reduction scenario, and by 9.4 (1.3–15.4) months in the 50% meat reduction scenario (Table 3). A major share (95%) of the estimated increased life expectancy was due to reduced mortality from CVD (ischaemic heart disease, ischaemic stroke, subarachnoid haemorrhage and intracerebral haemorrhage, combined) (Fig. 1). Furthermore, total YLG accumulated over 30 years from avoided deaths ranged between 8.4 (1.3–14.9) million and 10.9 (1.5–17.9) million in the 35% and 50% meat reduction scenarios, respectively. These gains were largely driven by reduced mortality from CVD (Fig. 2).

**Table 3:** Changes in life expectancy, years of life gained and incidence of disease, accumulated over 30 years, with a time to full effect of 10 years, for the 35% and 50% meat reduction scenarios, respectively

Outcome	35% meat reduction scenario^a^	50% meat reduction scenario^a^
LE in months (CI)	7.2 (1.1–12.9)	9.4 (1.3–15.4)
YLG^a^ in million (CI)	8.4 (1.3–14.9)	10.9 (1.5–17.9)
*Disease incidence*		
Colorectal cancer in nr (CI)	−47,046 (−2782 to −156,240)	−72,207 (−7841 to −225,156)
Type−2 diabetes in nr (CI)	−799,197 (−78,970 to −1,890,263)	−1,122,824 (−144,260 to −2,474,253)
CVD^a^ in nr (CI)	−2,545,353 (−363,482 to −4,538,914)	−3,299,217 (−428,209 to −5,406,950)

^a^Time to full effect of 10 years

LE = Life expectancy

YLG = Years of Life Gained

CVD = Cardiovascular disease

^a^Ischaemic heart disease, ischaemic stroke, subarachnoid haemorrhage and intracerebral haemorrhage, combined

**Fig. 3:**
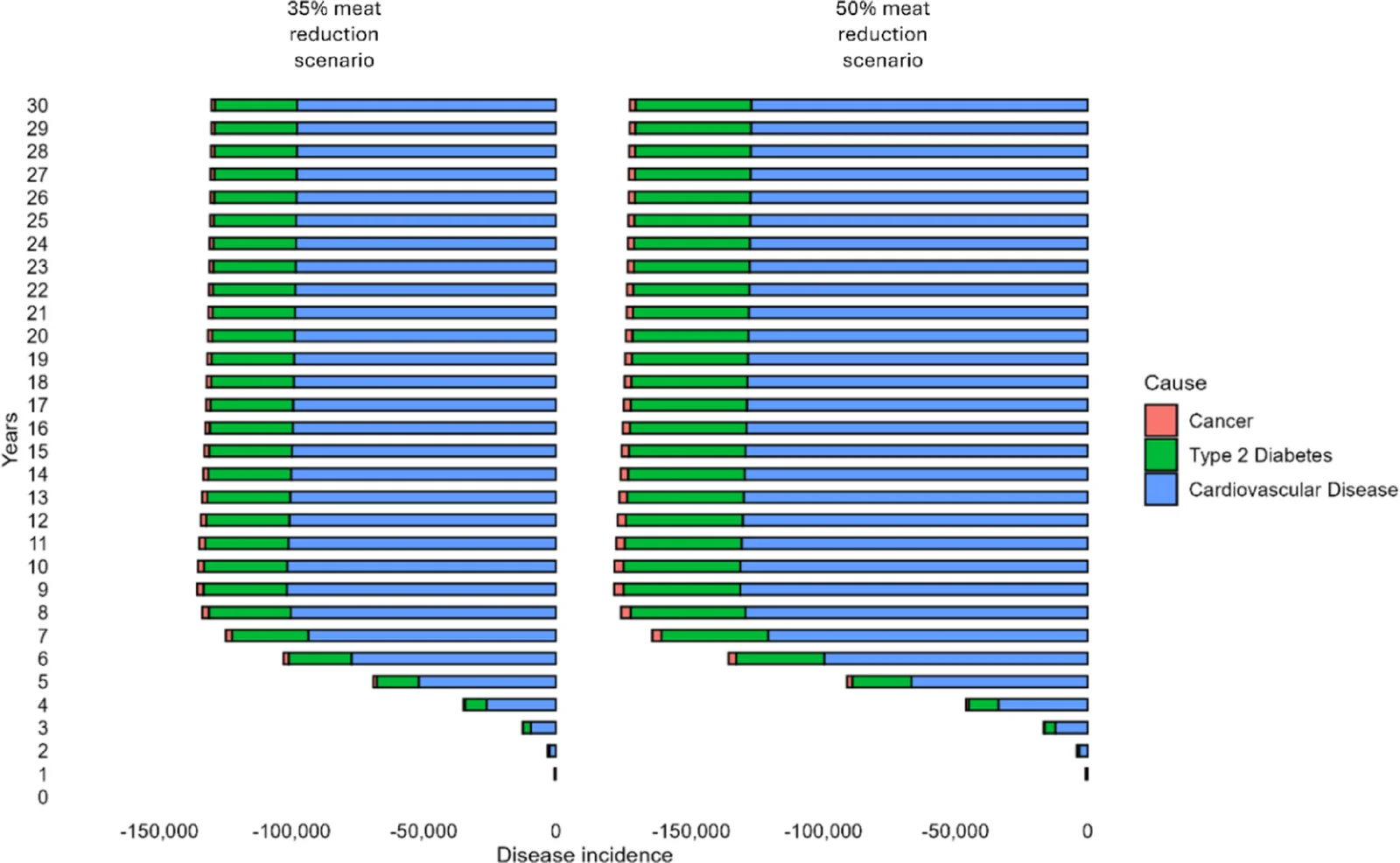
Share of changes in disease incidence by disease outcome, with a time to full effect of 10 years, for the 35% and 50% meat reduction scenarios, respectively

**Fig. 4:**
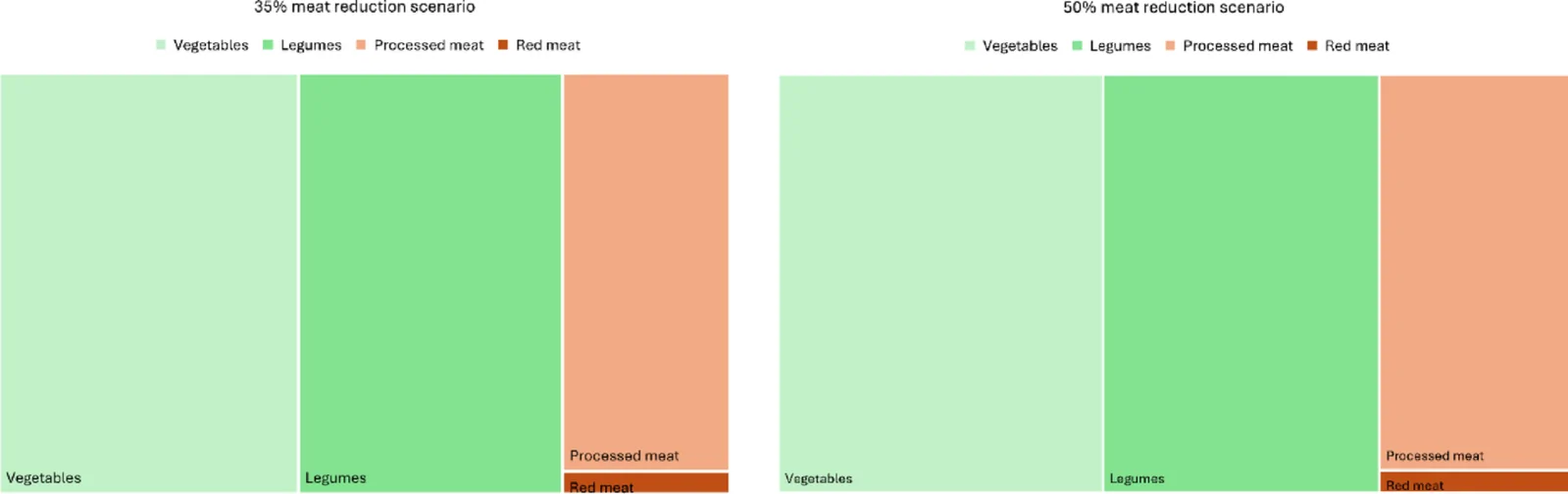
Share of health gains by dietary risk, with a time to full effect of 10 years, for the 35% and 50% meat reduction scenarios, respectively

Similar impacts were observed through reduced morbidity (Table 3). The largest share (approximately 75%) of reductions in new disease cases over 30 years was attributable to CVD, with an estimated 2.5 (0.4–4.5) and 3.3 (0.4–5.4) million fewer cases under the 35% and 50% meat reduction scenarios, respectively (Table 3, Fig. 3). This was followed by type 2 diabetes—which according to the GBD estimates is affected only by changes in red and processed meat consumption—with an estimated 0.8 (0.08–1.9) and 1.1 (0.1–2.5) million fewer cases under the two scenarios. (Table 3, Fig. 3). New cases of colorectal cancer over 30 years, also only impacted by the reduction in consumption of red and processed meat as per GBD estimates, constituted the lowest share of total reductions in disease incidence (Fig. 3) but still achieved reductions of up to 72 (7.8–225) thousand fewer cases in the 50% meat reduction scenario (Table 3). Overall, about three quarters of mortality gains could be attributed to changes in the consumption of vegetables and legumes (Fig. 4) with the same patterns for morbidity outcomes (no data shown).

The sensitivity analyses, which tested two alternative TTFEs (5 and 30y), showed similar health impacts, with slightly larger gains observed for TTFE 5 years and somewhat lower impacts for TTFE 30y compared to TTFE 10 years (Supplementary Table 2, Supplementary Figs. 2, 3, 4 and 5). More specifically, applying a TTFE of 5 years resulted in gains that were 7–10% larger than those obtained with the main model (TTFE of 10 years), depending on the scenario and outcome. In contrast, applying a TTFE of 30 years resulted in gains that were 28–35% smaller than those of the main model.

## Discussion

This modelling study demonstrated that achieving the UKCCC’s meat reduction targets through strong government-led interventions—specifically reductions in red and processed meat and their substitution with vegetables and legumes—could deliver substantial public health benefits, complementing previously reported environmental gains [22]. Over a 30-year horizon, the projected reduction in meat intake and corresponding increase in the consumption of vegetables and legumes was associated with a projected reduction of up to 4.5 million cases of chronic disease and gains of up to 9.4 months in population life expectancy in the UK under the assumptions of the model. Importantly, the health gains reported here are conditional on the behavioural dynamics simulated in the agent-based model and should be interpreted as exploratory scenario estimates rather than predictions of real-world outcomes. Given the broadly similar dietary patterns, chronic disease profiles, and shared climate mitigation commitments across many high-income European countries, these findings are likely to be informative for wider European policy discussions on sustainable diets, including those occurring at EU and WHO European Region levels. The main part of these gains can be attributed to reduced mortality and morbidity from CVD, which is the most prominent cause of death and disability in the UK and across Europe, and is also the disease category most strongly influenced by dietary risk factors [9]. These projected health gains align directly with national public health priorities outlined in recent UK strategies such as the NHS Long Term Plan [36] and the 10 Year Health Plan for England [37], which emphasise reducing the burden of cardiometabolic diseases. Similar priorities are reflected across European public health strategies, including WHO Europe targets for non-communicable disease reduction and EU-level prevention frameworks, where cardiovascular disease remains a leading cause of preventable mortality and a major contributor to rising health system expenditure. The anticipated gains are also likely to reduce the economic burden of cardiovascular disease, which is estimated to cost the wider UK economy £29 billion annually [38].

These results are consistent with—and extend—the findings of previous health modelling studies in the European Region and other high income settings, which have demonstrated that dietary shifts away from meat, particularly red and processed meats, are associated with substantial reductions in mortality and incidence of chronic diseases [14, 39–44]. They also tally with the findings of a recent policy modelling study [45] that found significant reductions in chronic disease mortality following the hypothetical implementation of a tax on meat at a global scale. Similarly, in a high-tax scenario in Germany [46], comparable reductions in processed meat intake were projected to yield decreases of 126,000 (146.5/100,000) cases of type−2 diabetes and 7200 (8.4/100,000) cases of colorectal cancer by year 10 of the simulation. These estimates are somewhat higher than the outcomes in our study at the same time point—31,556–44,285 (47.1–66.1/100,000) and 2292–3400 (3.4–5.1/100,000) cases respectively for type−2 diabetes and colorectal cancer across the two scenarios (no data shown)—which is likely attributable to methodological differences, particularly the use of different exposure–response functions as well as the absence of time-lag assumptions between changes in dietary exposure and subsequent disease impacts in their modelling approach. Moreover, Milner et al. [42] assessed the health impacts of multiple measures to achieve net zero GHGEs, including reductions in meat consumption according to the UKCCC’s targets, and reported similar trends in gains in YLG and life expectancy. However, their estimated health gains were somewhat lower than those identified in our study. Again, this difference may stem from methodological choices: their modelling of dietary change did not incorporate opinion-dynamics or social network-based substitutions and did not include reductions in processed meat consumption. Crucially, our results build on a novel agent-based modelling approach that explicitly explored the time required to achieve the 35% and 50% meat reduction targets. We found that these targets could in the most plausible scenario be reached in a shorter timeframe—approximately 5.2 years for reaching a 35% reduction and 8.1 years for a 50% reduction—compared to previous studies, which assumed the targets would only be met by 2030 and 2050, respectively. This suggests that earlier research may have underestimated the potential health gains associated with achieving these dietary targets. Comparing the magnitude of health impacts across studies is complicated by differences in scenario design, time horizon, baseline dietary patterns, and modelling approach. For example, a recent microsimulation study in the United States [16] estimated that a combined 30% reduction in both processed and unprocessed red meat would avert 1,073,400 (443/100,000), 382,400 (158/100,000), and 84,400 (35/100,000) occurrences of type 2 diabetes, cardiovascular disease, and colorectal cancer respectively over 10 years among US adults. These figures are broadly comparable in terms of absolute gram reductions to our 35% scenario for males, though somewhat larger than for females given lower baseline meat consumption in women. Over a 10-year period, our 35% scenario yields considerably fewer averted cases of type 2 diabetes (31,556; 47/100,000) and colorectal cancer (2292; 3.4/100,000), but a comparable number for cardiovascular disease (101,612; 152/100,000) (no data shown). The lower type 2 diabetes and colorectal cancer estimates in our study likely reflect the more conservative GBD-derived relative risks used here compared with the stronger associations from US cohort meta-analyses applied by Kennedy et al. Conversely, our cardiovascular disease estimate is comparable to Kennedy et al. despite their larger dietary changes and their omission of lag effects. This may reflect the additional protective effects of modelled increases in vegetable and legume consumption in our study—a substitution pathway not captured by Kennedy et al., who modelled dietary change without accounting for what replaces the meat. These comparisons illustrate how modelling choices can influence health impact estimates across outcomes, and more importantly highlight that the health benefits of reducing meat consumption are not solely determined by eating less meat, but also by what people choose to eat instead.

A large share of the modelled gains in our study was due to reduced mortality and morbidity from CVD. This tallies with findings from previous research [41, 43, 47] and reflects the fact that nearly all dietary risk factors considered in our model (the intake of vegetables, legumes and processed meat) are linked to cardiovascular outcomes, whereas cancers and type 2 diabetes are influenced by only two risk factors (red and processed meat). Furthermore, changes in CVD mortality and morbidity are largely impacted by changes in the intake of vegetables and legumes, for which the absolute dietary changes were considerably larger than those for red and processed meat.

A key strength is that the dietary changes underpinning our health impact modelling were informed by a novel agent-based opinion dynamics modelling approach, capturing more realistic patterns of dietary transition rather than assuming abrupt or uniform shifts. However, the model relies on stylised behavioural assumptions and synthetic social network structures. For example, peer influence is weighted by similarity in age and socioeconomic status, which captures homophily only partially: it does not extend to other socially relevant dimensions, such as shared dietary attitudes or geography, nor to the sparsity and clustering typical of real-world social networks, either of which could lead to an over- or underestimation of social contagion effects . Susceptibility to influence was further differentiated by baseline meat consumption rather than by income directly; given that consumption of some meats varies by income group in the UK [48], this may act as a partial proxy for socioeconomic differences in responsiveness, though it does not capture differential price elasticity by income group directly, as fiscal measures are modelled only as a uniform contributor to overall external influence. The health outputs should therefore be interpreted as conditional on these modelled trajectories rather than empirically observed dietary transitions. Despite these constraints, the modelling framework is readily adaptable beyond the UK context, since social norms, peer effects, and policy-driven food environment changes shape dietary behaviour similarly across other European countries. Combined with actual population estimates, underlying mortality and morbidity data, and robust epidemiological evidence on specific risk associations, this allows us to provide policy-relevant estimates of avoided chronic disease burden associated with large-scale dietary change scenarios. Sensitivity analyses around time lags to benefit realisation further address a critical area of uncertainty often overlooked in health impact modelling.

At the same time, our estimates carry some uncertainty due to the assumptions and data used in the model. First, while the model accounts for different underlying mortality and morbidity by age and sex, it assumes uniform dietary change and health response across the UK population. It does not capture the considerable heterogeneity and nuance in health status and dietary patterns across socioeconomic groups. While the underlying life table model already incorporates age- and sex-specific mortality and morbidity rates, results are presented for the UK population as a whole. Region-specific health impacts were not explored because the upstream agent-based simulation of dietary change was not disaggregated by region. Evidence suggests that health inequalities are widening in the UK, and that the burden of chronic disease remains disproportionately high among the most deprived households [49]. Our model does not reflect these gradients, and thus likely underestimates both the challenges and the opportunities for addressing health inequities in real-world settings. Furthermore, our model assumes that age- and sex-specific mortality and incidence rates remain constant throughout follow-up. This approach does not account for uncertainty in future trends—including the current increase in use of GLP-1 receptor agonists prescribed for weight loss and diabetes management in the UK [50]. These changes could lower chronic disease rates in the population and, consequently, alter the absolute health gains associated with dietary reductions in meat intake. Thus, our estimates should be interpreted in the context of uncertainty regarding future changes in both baseline risk and health determinants.

Our risk–benefit assessment was focused on the primary chronic diseases linked to red and processed meat, vegetables, and legumes, as classified in the GBD. We did not consider the risk of nutrient deficiencies or unintended consequences such as increased exposure to chemical contaminants such as heavy metals from higher vegetable and legume consumption. This leaves a considerable “black box” relating to nutritional adequacy, particularly in subpopulations or under sustained dietary transitions, and to food safety risks. Nutritional adequacy was not modelled because available relative risks for some nutrients are tightly linked to the food groups already included (e.g., fibre and vegetable/legume consumption) and including them separately would risk double-counting effects. Additionally, large reductions in meat intake may affect micronutrient adequacy, particularly for nutrients for which meat is a primary dietary source, such as iron and vitamin B12. While this represents a limitation of the current analysis, previous modelling work optimising diets towards healthier, more plant-forward patterns has shown that the resulting increase in iron-deficiency anaemia is real but modest relative to the chronic disease burden averted, with the anaemia-related loss representing under a third of the overall health gain [51]; the risk in a substitution scenario such as ours is therefore likely to be concentrated mainly in iron status, with comparatively little risk to vitamin B12 adequacy. Similar optimisation modelling research has demonstrated that nutritionally adequate diets (i.e. those meeting iron and vitamin b12 constraints) with substantially reduced meat intake and increased plant-based foods are achievable within current dietary patterns [52], suggesting that the health gains modelled here may not come at the expense of micronutrient adequacy. Chemical contaminant exposure risks were similarly not considered in the present analysis, as comprehensive estimation would require accounting for a wide range of chemicals, including heavy metals and pesticides, and would necessitate substantial modifications to the current modelling framework. While dose–response functions exist and have been used for many individual chemicals [53, 54], integrating them into our health impact model would require a considerably expanded approach. Future work could draw on risk–benefit assessment studies that incorporate multiple contaminants using alternative methodologies, allowing for a more complete evaluation of dietary risks. Furthermore, potential health effects arising from shifts in other parts of the diet, such as changes in refined carbohydrate, dairy, or total fat intake, were not captured, which may have influenced the magnitude of estimated health gains. This reflects the upstream agent-based opinion dynamics model [22], in which fiscal measures specifically incentivised vegetables, legumes, and meat alternatives as replacements for meat, leaving other dietary components unchanged by design.

Finally, the confidence intervals around our results were notably wide. This reflects an inherent feature of modelling dietary impacts: a combination of variation in dietary intake across the population and uncertainty arising from limitations in self-reported dietary data, for which no alternative high-resolution data exist. In the sensitivity analyses, ±1 standard deviation adjustments captured a range of modelled changes—from very small to very large—in consumption of red meat, processed meat, legumes, and vegetables. The substantially lower energy density of vegetables and legumes relative to meat means that isocaloric substitution necessarily requires larger increases in weight, particularly at the lower bound of these ranges; whether individuals would adopt dietary changes of this absolute scale remains an open empirical question, and these bounds are best interpreted as reflecting statistical variability in baseline dietary intake rather than distinct, equally plausible behavioural pathways. These wide intervals therefore represent both the heterogeneity in actual dietary patterns in the UK and the unavoidable imprecision of current dietary surveillance and reporting systems. As with all modelling studies of this type, results should be interpreted as indicative of potential, rather than guaranteed, population-level effects. They should also be complemented by research investigating the broader social and economic impacts of food policy changes in the UK, as such shifts could have wider unintended consequences [55].

This study set out to estimate the population health impacts of achieving the UKCCC's meat reduction targets—specifically a 35% and 50% reduction in red and processed meat consumption—through policy-driven dietary transitions in the UK. Our findings suggest that meeting these targets could be associated with substantial reductions in chronic disease burden and meaningful gains in population life expectancy, driven predominantly by reductions in cardiovascular mortality. While these estimates are exploratory and conditional on modelled behavioural trajectories, they provide a relevant indication of the potential scale of public health gains achievable through climate-aligned food policies, with implications extending beyond the UK to broader European efforts to reduce cardiometabolic disease burden and advance sustainable dietary transitions.

## Electronic supplementary material

Below is the link to the electronic supplementary material.

Supplementary file 1 (DOCX 19 kb)

## Data Availability

All data used in this study are publicly available from the original sources cited in the manuscript. No individual participant data were collected. The datasets used for the analyses, together with the data dictionary defining each variable, can be accessed from the original public repositories. The analytical code used to process the data and generate the results will be made available with publication. No additional, related documents are available. The analytical code will be available from the date of publication and can be obtained via request to the corresponding author (patricia.eustachio.colombo@ki.se). Access will be granted for academic and non-commercial research purposes, subject to appropriate citation of the original data sources.

## Abbreviations

CVD: Cardiovascular disease
CIs: Confidence intervals
GBD: Global Burden of disease
IHD: Ischaemic heart disease
NDNS: National diet and nutrition survey
RR: Relative risk
SD: Standard deviation
TTFE: Time to full effect
UK: United Kingdom
UKCCC: United Kingdom’s climate change committee
WHO: World Health Organisation
YLG: Years of life gained
