# Supplementary material for: Health co-benefits of sustainable dietary transitions to reduced red and processed meat intake in the United Kingdom: a modelling study
Source: Eur J Nutr. 2026 Sep 17;65(7):254. doi: 10.1007/s00394-026-04093-6 (PMC13586013; doi:10.1007/s00394-026-04093-6)
Supplement: Supplementary file 1 [file 394_2026_4093_MOESM1_ESM.docx]

**Supplementary Information**

**Health co-benefits of modelled sustainable dietary transitions towards reduced red and processed meat intake in the United Kingdom**

**Table of contents**

[**Materials and methods**	2](#_Toc233730462)

[**Handling input data and relative risks**	2](#_Toc233730463)

[**Assumptions and lags implemented in the modelling**	2](#_Toc233730464)

[**Supplementary Table 1.** Main assumptions of the health impact model.	3](#_Toc233730465)

[**Supplementary Figure 1.** Time lag functions applied uniformly to all six modelled disease outcomes, under the three time-to-full-effect (TTFE) scenarios tested (5, 10, and 30 years). Each curve represents the proportion of the full relative risk reduction assumed to be realised at a given number of years following the dietary change	4](#_Toc233730466)

[**Results**	5](#_Toc233730467)

[**Supplementary Table 2**. Changes in life expectancy, years of life gained and incidence of disease, accumulated over 30 years, 10 years into the health impact projection for the 35% and 50% meat reduction scenarios, respectively.	5](#_Toc233730468)

[**Supplementary Figure 2**. Share of changes in years of life gained by disease outcome 5 years into the health impact projection for the 35% and 50% meat reduction scenarios, respectively.	6](#_Toc233730469)

[**Supplementary Figure 3**. Share of changes in years of life gained by disease outcome 30 years into the health impact projection for the 35% and 50% meat reduction scenarios, respectively.	7](#_Toc233730470)

[**Supplementary Figure 4.** Share of changes in disease incidence by disease outcome 5 years into the health impact projection for the 35% and 50% meat reduction scenarios, respectively.	8](#_Toc233730471)

[**Supplementary Figure 5.** Share of changes in disease incidence by disease outcome 30 years into the health impact projection for the 35% and 50% meat reduction scenarios, respectively.	9](#_Toc233730472)

[**References**	10](#_Toc233730473)

# **Materials and methods**

## **Handling input data and relative risks**

The disease-specific mortality and morbidity data were only available by 5-year age ranges and not in single-year-of-age format. Therefore, the rate for each 1-year age interval was interpolated using monotonic cubic spline interpolation using the programme SRS splines, available as an add-in to Excel (22).

Relative risks from the GBD which were expressed in terms of a harmful risk factor (e.g. “diet high in sodium/salt”) were inverted to create relative risks for a positive change in diet. The GBD provides relative risks for females and males combined, by 5-year age ranges, starting from the age of 25. The relative risks for each risk, outcome, and 5-year age range were weighted according to the age distribution in the UK population to create one single relative risk for each risk-outcome pair. Ages from 18-25 were assigned the same relative risk as the 25-29 age group, although the baseline mortality from the included dietary risks was almost non-existent in the UK population aged 18-25. For the analysis, the exposure-response functions were assumed to be log-linear. The change in mortality risk *R* associated with a modelled change in dietary exposure *E* was calculated as:

Where RR_∆E_ is the relative risk associated with a fixed change in dietary risk exposure (∆E) derived from the GBD.

## **Assumptions and lags implemented in the modelling**

A full list of assumptions for the health impact modelling, as well as where to find these in the text, may be found in Supplementary Table 1. We assumed that shifts in consumption patterns were adopted instantaneously, and that underlying mortality and incidence rates remained constant throughout the follow-up period. Furthermore, the model projected a consistent number of new live births into the future. In cases where several dietary exposures affected the same disease, the risks were multiplied together as done previously (23). To account for the time required for dietary changes to manifest as health impacts, we integrated time lags based on established epidemiological evidence (26,27) and a previously applied modelling approach (28). Specifically, we assumed that the time to full effect (TTFE) on all six modelled disease outcomes (ischaemic heart disease, ischaemic stroke, intracerebral haemorrhage, subarachnoid haemorrhage, type 2 diabetes, and colorectal cancer) was approximately 10 years in the main analyses. These time lags were implemented using time-varying functions derived from cumulative distribution functions of normally distributed variables, resulting in s-shaped curves (Supplementary Figures 1-3) to represent the gradual onset of health benefits. The same lag function shape, rescaled to the relevant TTFE, was applied uniformly across all modelled disease outcomes, as a simplifying assumption representing a compromise between the comparatively rapid risk reduction reported for cardiovascular disease and the longer latency typically associated with cancer outcomes. As this curve represents a deterministic structural assumption rather than a statistically estimated function, a conventional sampling-based confidence interval is not applicable; instead, the sensitivity of our results to this assumption is assessed directly by varying the TTFE itself (5, 10, and 30 years; Supplementary Figure 1). Further details regarding the precise implementation of these time lags have been previously described (28).

## **Supplementary Table 1.** Main assumptions of the health impact model.

|  | **Main assumptions** | **Place in text** |
| --- | --- | --- |
| 1 | Relative risks for each risk, outcome, and 5-year age range were weighted according to the age distribution in the UK population | Page 3 Supplementary Materials |
| 2 | Relative risks from the GBD were inverted to create relative risks for a positive change in diet | Page 5 main text, page 3 Supplementary Materials |
| 3 | Disease-specific mortality and morbidity data was interpolated using monotonic cubic spline interpolation to get the rate for each 1-year age interval | Page 3 Supplementary Materials |
| 4 | Changes vegetables, legumes, red meat, and processed meat consumption were assumed to be adopted instantly. | Page 3 Supplementary Materials |
| 5 | Underlying mortality and incidence rates remained constant for the duration of follow-up | Page 3 Supplementary Materials |
| 7 | The same number of new live births into the future were assumed | Page 3 Supplementary Materials |
| 8 | The effects on all disease outcomes were assumed to reach their maximum impact after 10 years in the main analyses, and after 5 and 30 years, respectively, in the sensitivity analyses | Pages 6-7 main text and page 3 Supplementary Materials |
| 9 | To account for the time delays between dietary changes and health outcomes, time-varying functions based on cumulative distribution curves were used | Page 3-4 Supplementary Materials |
| 10 | In cases where several dietary exposures affected the same disease, the risks were multiplied together to avoid double counting. | Page 9 main text |

## **Supplementary Figure 1.** Time lag functions applied uniformly to all six modelled disease outcomes, under the three time-to-full-effect (TTFE) scenarios tested (5, 10, and 30 years). Each curve represents the proportion of the full relative risk reduction assumed to be realised at a given number of years following the dietary change

# **Results**

## **Supplementary Table 2**. Changes in life expectancy, years of life gained and incidence of disease, accumulated over 30 years, with a time to full effect of 5 and 30 years, for the 35% and 50% meat reduction scenarios, respectively.

| **Outcome** | **35% meat reduction scenario^a^** | **50% meat reduction scenario^b^** | **35% meat reduction scenario^a^** | **50% meat reduction scenario^b^** |
| --- | --- | --- | --- | --- |
| LE (months) | 7·22 (1·08 to 12·9) | 9·39 (1·3 to 15·42) | 7·22 (1·08 to 12·9) | 9·39 (1·3 to 15·42) |
| YLG^a^ (million) | 9·5 (1·43 to 16·92) | 12·34 (1·71 to 20·22) | 6·19 (0·93 to 11·04) | 8·04 (1·12 to 13·2) |
| Disease incidence |  |  |  |  |
| Colorectal dancer | -50,495 (-2,864 to -169,981) | -77,859 (-8,398 to -245,670) | -33,909 (-2,328 to -106,593) | -51,102 (-5,703 to -151,723) |
| Type-2 diabetes | -879,798 (-86,887 to -2,081,616) | -1,236,212 (-158,794 to -2,725,018) | -518,839 (-51,397 to -1,225,182) | -728,526 (-93,699 to -1,602,879) |
| CVD^a^ | -2,799,015 (-399,282 to -4,995,682) | -3,629,187 (-470,374 to -5,953,242) | -1,659,300 (-238,227 to -2,945,476) | -2,147,166 (-280,672 to -3,502,116) |

^a^Time to full effect of 5 years.

^b^Time to full effect of 30 years.

LE = Life expectancy.

YLG = Years of Life Gained.

CVD = Cardiovascular disease.

^a^Ischemic heart disease, ischemic stroke, subarachnoid haemorrhage and intracerebral haemorrhage, combined

## **Supplementary Figure 2**. Share of changes in years of life gained by disease outcome, with a time to full effect of 5 years, for the 35% and 50% meat reduction scenarios, respectively.

## **Supplementary Figure 3**. Share of changes in years of life gained by disease outcome, with a time to full effect of 30 years, for the 35% and 50% meat reduction scenarios, respectively.

## **Supplementary Figure 4.** Share of changes in disease incidence by disease outcome, with a time to full effect of 5 years, for the 35% and 50% meat reduction scenarios, respectively.

## **Supplementary Figure 5.** Share of changes in disease incidence by disease outcome, with a time to full effect of 30 years, for the 35% and 50% meat reduction scenarios, respectively.

# **References**

1.	Srs1 Software LLC. SRS1 cubic spline for Microsoft Excel v2.5 for Windows. Massachusetts: Srs1 Software LLC; 2015.

2.	Harashima E, Nakagawa Y, Urata G, et al. Time-lag estimate between dietary intake and breast cancer mortality in Japan. Asia Pac J Clin Nutr. 2007;16(1):193–8.

3.	Capewell S, O’Flaherty M. Can dietary changes rapidly decrease cardiovascular mortality rates? Eur Heart J. 2011 May 2;32(10):1187–9.

4.	Milner J, Green R, Dangour AD, et al. Health effects of adopting low greenhouse gas emission diets in the UK. BMJ Open. 2015 Apr 30;5(4):e007364.

5.	Hendriksen MAH, van Raaij JMA, Geleijnse JM, et al. Health Gain by Salt Reduction in Europe: A Modelling Study. Gorlova OY, editor. PLOS ONE. 2015 Mar 31;10(3):e0118873.

6.	Vos T, Lim SS, Abbafati C, et al. Global burden of 369 diseases and injuries in 204 countries and territories, 1990–2019: a systematic analysis for the Global Burden of Disease Study 2019. The Lancet. 2020 Oct;396(10258):1204–22.

7.	Bandy LK, Scarborough P, Harrington RA, et al. Reductions in sugar sales from soft drinks in the UK from 2015 to 2018. BMC Med. 2020 Dec;18(1):20.
